# Advancement in Current Therapeutic Modalities in Postpartum Cardiomyopathy

**DOI:** 10.7759/cureus.22813

**Published:** 2022-03-03

**Authors:** Kamlesh Chaudhari, Mahak Choudhary, Kushagra Chaudhari, Neeta Verma, Sunil Kumar, Sparsh Madaan, Dhruv Talwar

**Affiliations:** 1 Department of Obstetrics and Gynaecology, Jawaharlal Nehru Medical College, Datta Meghe Institute of Medical Sciences (Deemed to be University), Wardha, IND; 2 Department of Neonatology, King Edward Memorial Hospital and Seth Gordhandas Sunderdas Medical College, Mumbai, IND; 3 Department of Anaesthesiology, Jawaharlal Nehru Medical College, Datta Meghe Institute of Medical Sciences (Deemed to be University), Wardha, IND; 4 Department of Medicine, Jawaharlal Nehru Medical College, Datta Meghe Institute of Medical Sciences (Deemed to be University), Wardha, IND

**Keywords:** cardiac pathologies, peripartum complications, bromocriptine, feto maternal morbidity, peripartum cardiomyopathy

## Abstract

Pregnancy is considered one of the most delicate conditions for the woman and her fetus, rendering physiological stress on her body. Sometimes, this leads to unwelcomed incidences of certain systemic disorders which further complicate the course of pregnancy. Cardiovascular conditions associated with pregnancy have major morbidity amongst the general population. Peripartum cardiomyopathy, one such condition associated with cardiac dysfunction during pregnancy, is one of the major causes of increased morbidity and mortality in pregnant women. It has been the leading cause of non-obstetric maternal mortality. Due to the stress on the cardiovascular system, further functioning of the body in the milieu gets compromised and thus, the occurrence of fetomaternal mortality is not rare in the prognosis of this condition. Certain studies have noted not only familial but also geographical variations in the prevalence of peripartum cardiomyopathy in certain areas. Although the occurrence of the condition is quite common, there still needs to be a better understanding of this topic for avoiding the abysmal prognosis of this pathology. A peculiar presentation on the electrogram is needed to make an accurate diagnosis of the condition. The therapeutic options of this condition, particularly incline towards medical management. Various new drugs have been formulated and are in clinical trials for testing their effectiveness. Bromocriptine therapy, along with the neoadjuvant combination of anticoagulant drugs and non-pharmacological measures, makes a good treatment regimen that helps avert the progressive pathology. In this article, we discuss the knowledge regarding the etiology, factors contributing to the severity, pathogenesis, treatment options, and the particular outcomes of the therapy.

## Introduction and background

Pregnancy is associated with various multi-systemic disorders, however, the leading systemic disorder amongst them is cardiovascular disorder. Nearly 5% of cardiovascular diseases do not amount to mortality with a positive outcome, however, peripartum cardiomyopathy is one such condition where a high incidence of fetomaternal mortality is seen [1,2]. Although peripartum cardiomyopathy is associated with high mortality rates and is often misdiagnosed, the clear knowledge regarding its etiology, pathogenesis, and management has not been discussed in depth. Peripartum cardiomyopathy is often associated with other cardiac events such as left ventricular systolic dysfunction. This systolic dysfunction leads to vascular disturbances and the effective blood flow to the growing fetus could be compromised.

Amongst the various cardiovascular disorders affecting fetal health during pregnancy, peripartum cardiomyopathy depicts a rather varied course of presentation. The research on this pathology has been limited due to the ambiguous medical presentation. Often this pathology is seen affecting the primigravida with no previous history of any cardiac abnormalities [3]. Due to the advancement in medical sciences, the prognosis of this disease is generally good, but in severe cases, it might result in mortality [4,5].

Postpartum cardiomyopathy, also known as peripartum cardiomyopathy, is defined as new-onset heart failure occurring during the last month of gestation to the first five months following delivery with no determinable cause [6]. Currently, the diagnostic criteria for peripartum cardiomyopathy include [6]: 1) Cardiac failure which occurs in a previously healthy woman during the last month of pregnancy or during the first five months following delivery. 2) Cardiac failure with no determinable etiology. 3) Absence of any demonstrable cardiac disease prior to the last month of pregnancy. 4) Echocardiography showing evidence of reduced left ventricular systolic function.

Methodology

Protocol and Registration

The review protocol was registered at Jawaharlal Nehru Medical College. The review was limited to the advanced treatment modalities in the prognosis of peripartum cardiomyopathy.

Search Strategy

We used Medline, EMBASE, and the Cochrane Library to conduct our research. PPCM OR cardiac abnormalities in pregnancy OR treatment OR bromocriptine OR electrocardiography OR ejection fraction were used as search terms. Only English-language publications were found in the search results. Additional relevant research was found by individually searching the sources of the included publications.

Study Selection

**Figure 1:**
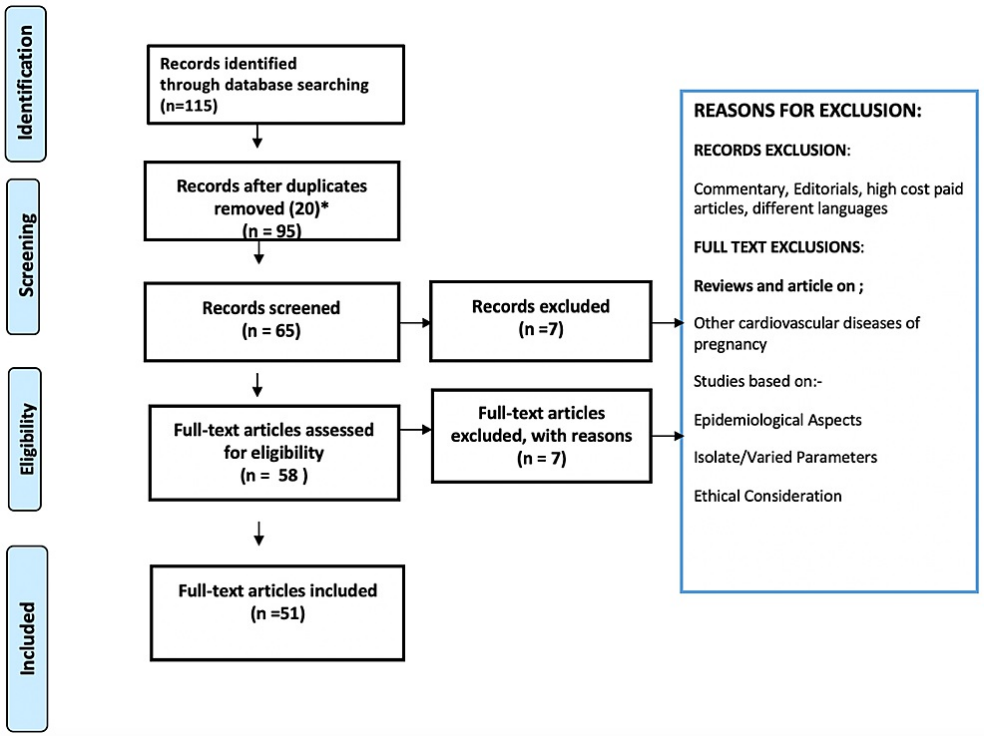
Depicting the Preferred Reporting Items for Systematic Reviews and Meta-Analyses (PRISMA) method for review of literature in the present review

In this literature review, the Preferred Reporting Items for Systematic Reviews and Meta-Analyses (PRISMA) [7] method was used. We included certain observational studies, case reports, reviews, and various original scientific research papers. We included studies that depicted a co-relation between peripartum cardiomyopathy and various treatment modalities. Only the most recent articles were considered for this review. A flowchart showing the methodology and study selection is presented in Figure 1.

## Review

Review results

Pathophysiology

The possible pathophysiology of peripartum cardiomyopathy continues to puzzle clinicians throughout the globe. Endocardial biopsy testing has shown comparable viral serologies between patients of peripartum cardiomyopathy and control groups which disregards the popular theory of viral etiology [8]. With the development of magnetic resonance imaging, there has been the demonstration of myocarditis suggestive features in patients of peripartum cardiomyopathy [7]. Also, the changes in the pregnancy lead to an excess of hemodynamic stress to the heart which causes synergistic action over the heart to develop cardiomyopathy. Currently, the two-hit model of peripartum cardiomyopathy is considered to be appropriate with a vascular humoral insult along with genetic predisposition [9]. Multiple genetic targets play an essential role in developing peripartum cardiomyopathy including titin protein (TTN), BCL2-associated athanogene 3 (BAG3), parathyroid hormone-related protein (PTHLH), and peroxisome proliferator-activated receptor-gamma coactivator 1-alpha (PGC-1α) [9,10]. 

Clinical Presentation and Examination

Clinical manifestation of peripartum cardiomyopathy might masquerade as any other systolic heart failure [11]. The clinical spectrum may vary from mild symptoms to rapid progression to end-stage heart failure and ultimately death. However, it is common for the symptoms of peripartum cardiomyopathy to subside leading to spontaneous recovery. Majority of the patients present in the first four months following childbirth. Peripartum cardiomyopathy may present in the form of dyspnoea, orthopnoea, cough which is present particularly while the patient lies down, palpitations, swelling of the legs, and dizziness. Hepatic congestion may lead to discomfort in the abdomen.

On examination, signs of heart failure may be evident which consists of sinus tachycardia, a raised jugular venous pressure, and bilateral crepitations on auscultation of the lungs [9]. Other findings which may be present are third heart sound and a displaced apex beat. The report submitted by 2019 MBRRACE highlights that persistent tachycardia, orthopnoea, chest pain, and tachypnoea are red flag signs which indicate a cardiac pathology and should be thoroughly investigated [12]. It is important to note that the presenting features of peripartum cardiomyopathy may be indistinguishable from physiological symptoms of pregnancy and postpartum period making the diagnosis a difficult task.

Differential Diagnosis

Differential diagnosis of peripartum cardiomyopathy includes pre-existing cardiomyopathy as well as valvular heart disease. The commonest valvular heart diseases to be encountered in pregnancy are mitral stenosis and aortic stenosis [9]. During the late second trimester, cardiac output, as well as the plasma volume increases by 50% followed by a plateau for the rest of the pregnancy hence, the patients with pre-existing cardiomyopathy or valvular heart disease, tend to become symptomatic earlier in the course of pregnancy when compared with patients having peripartum cardiomyopathy. Atherosclerotic plaque rupture or dissection of the coronary artery may result in myocardial infarction during the early postpartum period. Other conditions which can have similar presentations are pulmonary embolism and amniotic fluid embolism. The risk of pulmonary embolism is five to ten times more during the pregnancy and postpartum period whereas amniotic fluid embolism which is characterized by respiratory failure and shock during pregnancy or immediately after childbirth is less frequently seen. To further narrow down the diagnosis of peripartum cardiomyopathy, the American Heart Association has drafted clinical guidelines which state the onset of symptoms of heart failure prepartum or a few days postpartum, alongside the presence of left ventricular dysfunction would indicate the diagnosis of peripartum cardiomyopathy [13]. 

Investigations

Peripartum cardiomyopathy is diagnosed following a thorough workup after excluding all other cardiac as well as non-cardiac causes of heart failure [14,15]. Strict vigilance by the treating clinicians along with prompt work up aids in timely diagnosis. An electrocardiogram is often the primary investigation that is advised even on the grass route levels of health care for evaluation of any patient arriving with symptoms and signs of heart failure. It is a simple but highly informative test to evaluate cardiac function. Although there are no characteristic findings in the electrocardiogram to suggest peripartum cardiomyopathy, there may be certain nonspecific ST-segment changes along with abnormalities in the T wave [16]. A common problem in the evaluation of the electrocardiogram is the absence of previous findings to evaluate the differences which might have occurred over time. Although not a diagnostic test for peripartum cardiomyopathy, an electrocardiogram is definitely an important test to detect pulmonary embolism and acute myocardial ischemia which might have similar clinical presentations [17]. Inflammatory markers such as c-reactive protein and leucocyte count can be raised whereas hemoglobin levels can be decreased; however, these are non-specific for peripartum cardiomyopathy. Brain natriuretic peptide (BNP) or N-terminal pro-brain natriuretic peptide (NT-proBNP) is a well-established marker for heart failure and is raised in peripartum cardiomyopathy [18]. Normal ranges are values less than 100 pg/ml for BNP and less than 300 pg/ml for NT-proBNP respectively [19]. It is important to note that troponin T which is a cardiac enzyme and a marker for injury to the cardiac myocyte is often in the normal range for patients of peripartum cardiomyopathy. A chest X-ray may show cardiomegaly with pulmonary edema or pleural effusion along with alveolar shadowing and septal lines [20]. Echocardiography has emerged as the most important and non-invasive investigation to evaluate left ventricular ejection fraction. An important criterion to define peripartum cardiomyopathy is a left ventricular ejection fraction of less than 45% [9].

Echocardiography is also useful to provide an idea about the prognosis of peripartum cardiomyopathy. Right ventricular dysfunction which can be seen on echocardiography is associated with poor outcomes [11]. Also, the best tools to predict the recovery for peripartum cardiomyopathy are left ventricular ejection fraction and size upon the diagnosis of peripartum cardiomyopathy [9]. Left ventricular end-diastolic diameter >6 cm and left ventricular ejection fraction of less than 30% are associated with decreased chances of recovery and increased probability of requiring mechanical support, cardiac transplant, or death [9]. Cardiac magnetic resonance imaging is a highly useful imaging modality for cardiac assessment. It assesses the systolic function of the volume of the cardiac chamber [21]. It is highly sensitive to detect a thrombus that is intracardiac and missed on echocardiography. Fibrosis, necrosis, leakage of capillaries, hyperemia, and interstitial as well as intracellular injury can also be picked up on cardiac magnetic resonance imaging indicating tissue injury [21]. A drawback for this modality remains the use of gadolinium contrast which obstructs it from becoming an initial investigation in pregnancy and the high cost associated with it makes it a less used investigation tool especially in rural areas or in a limited resource setting. According to the European Society of Radiology, gadolinium contrast in pregnancy should only be given if it is absolutely necessary. However, breastfeeding should not be stopped following the use of gadolinium contrast [9]. Endomyocardial biopsy (EMB) is rarely performed investigation done only when there is extreme uncertainty of diagnosis in order to exclude other pathologies which might result in left ventricular dysfunction [22].

Medical management

Due to the sparsity of data from studies performed exclusively in women with peripartum cardiomyopathy, treatment guidelines are usually adapted from other forms of heart failure with reduced ejection fraction or through expert opinions. Standard treatment for heart failure with reduced ejection fraction should be practiced in cases of peripartum cardiomyopathy with special attention devoted to avoiding adverse effects on the fetus in patients who are still pregnant at the time of diagnosis. The main aspect of volume management remains sodium restriction along with the initiation of a loop diuretic for the management of peripheral as well as pulmonary edema [23]. Extra care should be provided while using loop diuretics in order to avoid the ill effects of over-diuresis such as hypotension during pregnancy which might cause uterine hypoperfusion. If the hemodynamic parameters permit the use of β blockers, preferably a selective β1 blocker should be prescribed which includes metoprolol tartrate in order to prevent uterine contractions which are mediated through the β2 receptors [24].

Angiotensin convertase enzyme inhibitors, as well as angiotensin receptor blockers, are contraindicated during pregnancy due to their teratogenic effects which have been reported through various studies. However, certain angiotensin-converting enzyme inhibitors are compatible with breastfeeding [25]. Sacubitril-valsartan which is an angiotensin receptor and neprilysin inhibitor widely used in the management of heart failure is also contraindicated in pregnancy with a lack of data regarding its effect on breastfeeding.

The RALES and EMPHASIS-HF trials have concluded that mineralocorticoid receptor antagonists are beneficial if used in patients suffering from New York Heart Association II-IV heart failure and a left ventricle ejection fraction of less than 35% [26]. However, these agents are also contraindicated in pregnancy owing to their androgenic effects but can be used in patients who are breastfeeding.

Vasodilator therapy in the form of hydralazine combined with nitrates can be prescribed for the management of peripartum cardiomyopathy through the reduction of afterload. Digoxin can also be used safely during pregnancy [27]. As warfarin is contraindicated in pregnancy and there is no data present on the use of direct oral anticoagulants, low molecular weight heparin can be used for anticoagulation if required. Peripartum cardiomyopathy may be complicated with thromboembolism commonly. The American Heart Association recommends anticoagulation in patients with an ejection fraction of less than 30% [28]. Some expert panels have recommended the use of anticoagulants in all patients with peripartum cardiomyopathy till eight weeks following childbirth [8]. During the event of an arrhythmia which can not be controlled through the use of β-blockers, drugs such as procainamide, verapamil and flecainide might be used [9]. However, there is insufficient data on their safety profile.

Postpartum Breastfeeding of Hemodynamically Stable Patients

During the postpartum period, several of the angiotensin convertase enzyme inhibitors, angiotensin receptor blockers, and mineralocorticoid receptor antagonists are compatible with breastfeeding. Beta-blockers, nitrates, and hydralazine may be continued, however, it is preferable to initiate angiotensin convertase enzyme inhibitors or angiotensin receptor blockers [9]. Low molecular weight heparin may be continued; however, warfarin can also be given during the breastfeeding postpartum period.

Haemodynamically Unstable Patients

There is a need for prompt aggressive therapy in hemodynamically unstable patients. Initial management includes admission to the intensive care unit followed by reduction of pre-load through the administration of intravenous diuretics. Nitrates can be administered safely in patients with a systolic blood pressure above 110 mmHg leading to vasodilatation [22]. Providing adequate oxygenation is of utmost importance, with a SpO2 of more than 95% in a propped-up position [22]. Timely usage of non-invasive ventilation and continuous positive airway pressure might decrease the need for intubation. Mechanical ventilation following intubation is indicated in patients with refractory hypoxia.

Circulatory Support With Inotropic and/or Vasopressor Therapy

Cardiac shock is a state of inadequate cardiac output leading to decreased perfusion of the end organs resulting from cardiac dysfunction. In such cases, there is a need for circulatory stabilization in order to prevent permanent damage to the end organs. This stabilization is achieved through the use of vasopressors and inotropes. Adrenaline and dobutamine increase the oxygen demand of the myocardium and are hence not used frequently in peripartum cardiomyopathy. A calcium sensitizing agent named levosimendan has recently been used as an inotropic agent in peripartum cardiomyopathy in order to provide circulatory support. A study of 28 patients suffering from cardiogenic shock, out of which eight patients had peripartum cardiomyopathy, reported that levosimendan improved the systolic function as well as hemodynamics in the peripartum cardiomyopathy subset of the study [29]. However, a randomized control trial conducted in 24 patients suffering from peripartum cardiomyopathy reported no improvement in left ventricular ejection fraction or survival rate following the use of levosimendan over standard treatment protocol for heart failure [30]. It is important for the multi-specialty team managing a case of peripartum cardiomyopathy to decide the timing for delivery. Maternal safety is given the first preference in such cases and a decision for urgent delivery irrespective of the duration of pregnancy should be made in an unstable patient.

Mechanical Circulatory Support

In patients with heart failure with reduced ejection fraction where the inotropic support fails to provide hemodynamic stability, mechanical circulatory support is indicated [30]. A major drawback of this modality is its non-availability in certain peripheral centers and the high cost associated with it. Intraaortic balloon pump and intraventricular pump which may be inserted using access from a peripheral artery such as the femoral artery may be used to provide short-term therapy initially [31]. There is an augmentation of circulation by the intra aortic balloon pump leading to improvement of coronary flow; whereas, the cardiac output is maintained by the use of an intraventricular pump. None of these two options can affect oxygenation. In cases of refractory pulmonary dysfunction, the patients may benefit from the use of extracorporeal membrane oxygenation (ECMO) which again has limited availability [22]. In order to bridge the time period till recovery or till a definitive treatment such as a cardiac transplant is performed, devices such as the biventricular assist or the left ventricular assist may be used. Patients undergoing cardiac transplants for peripartum cardiomyopathy have poor outcomes when compared to cardiac transplants for other indications with higher rates of mortality, graft rejection, and need for repeat transplantations [22]. However, in a study conducted by Bouabdallaoui et al., which studied the long-term outcome for heart transplantation in peripartum cardiomyopathy, it was concluded that long-term outcomes for patients with peripartum cardiomyopathy were favorable [32]. A total of 14 cardiac transplants were performed for peripartum cardiomyopathy out of a single-center series of 1938 cardiac transplants. Analysis was done for peripartum cardiomyopathy patients with 28 sex-matched controls and it was found that during a median follow-up of 7.7 years, 21.5% mortality was reported in the peripartum cardiomyopathy group whereas 46.5% mortality was reported in the control group [32]. Defibrillation is considered a viable option for the management of cardiac arrest and serious arrhythmia in patients with peripartum cardiomyopathy during pregnancy as well as breastfeeding. In the case of non-emergent cardioversion, special care for fetal monitoring should be ensured to detect secondary arrhythmias in the fetus [10].

Advanced Heart Failure Therapy

The implantable cardiac defibrillator was initially used for the prevention of arrhythmias in heart failure with reduced ejection fraction; however, with the advancement in medical therapy, these devices are no longer indicated. The American Heart Association has suggested the use of these devices in peripartum cardiomyopathy with severe impairment in order to bridge the time period till intracardiac device implantation or till recovery of ejection fraction for a period of three to six months [9].

Pregnancy-specific considerations

For patients who develop peripartum cardiomyopathy during the antepartum period, a multi-specialty team should be appointed to draft an individual care plan for the patient and to decide the timing as well as the preferred mode of delivery. Patients who respond to medical management may continue the pregnancy in the presence of close monitoring; however, cesarean delivery should be considered in patients with acute heart failure or in the presence of other obstetric indications [32]. Dense epidural anesthesia can be used to minimize the hemodynamic instability of labor along with assistance provided in the form of vacuum or forceps in the second stage of labor [13]. Misoprostol and carboprost (PGF2α) are reserved as a second-line management option in order to mitigate the risk of hemorrhage [23]. Heart failure due to medication-induced hypertension can be a side effect of oxytocin analogs and ergometrine, hence, they are not used in patients with peripartum cardiomyopathy.

Recent advances in the management of peripartum cardiomyopathy

Prolactin Inhibition

There have been new findings in the research related to pathogenesis behind peripartum cardiomyopathy making way for prolactin inhibition as a treatment modality. Lactation is inhibited by bromocriptine and cabergoline as they are dopamine D2 agonists and they also suppress the production of prolactin [33]. Ischemic stroke and myocardial infarction have been reported as part of thrombotic complications arising from the use of bromocriptine; this was reported when it was being used to suppress lactation which resulted in its quick withdrawal of the United States approval in 1995 [13]. In a randomized controlled trial conducted in peripartum cardiomyopathy patients in South Africa, a total of 20 patients were enrolled who were administered bromocriptine and it was reported that these patients had better improvement in the left ventricular ejection fraction [34]. The mean left ventricular ejection fraction improved from 27% at the beginning to 58% following bromocriptine administration versus 36% in the control group with a p-value of 0.012. However, this trial had a few drawbacks in the form of a small sample size and a high mortality rate in the control group. In an observational study conducted in Germany, 72% of the patients had improved following bromocriptine administration compared to 35% who did not experience any improvement [35]. A recent randomized controlled trial conducted in Germany concluded that 63 patients with peripartum cardiomyopathy showed no difference in the improvement of left ventricular ejection fraction when compared for one week versus an eight-week regimen of bromocriptine [36]. No morality was reported and none of the patients required a left ventricular assist device. However, one case of peripheral arterial thrombosis and a total of two cases of venous thromboembolism were reported even with the use of prophylactic anticoagulation. Prolactin inhibition, as a treatment option for peripartum cardiomyopathy, remains a topic of controversy worldwide with differences of opinion regarding its safety and its placement in the standard treatment protocol. Currently, the initiation of bromocriptine in peripartum cardiomyopathy may be justified if the left ventricular ejection fraction is less than 25% or there is a hemodynamic compromise in the form of cardiogenic shock [25]. It should be given at a dose of 2.5 mg twice daily for the duration of two weeks followed by 2.5 mg once daily for six weeks in such cases along with anticoagulation. As breastfeeding is extremely beneficial to maternal health as well as the child’s health and given the increased risk of thrombotic complications associated with bromocriptine, further studies are indeed required before establishing it as a routine treatment option for peripartum cardiomyopathy.

Antisense Therapy Against MicroRNA-146a

The antisense therapy against microRNA-146a has a theoretical benefit over other treatment modalities; when used, it authorizes the continuation of lactation in patients who are breastfeeding. However, this treatment option is yet to be tested in human subjects and has only been studied in mouse models. It was observed that the administration of antisense oligonucleotides against miR-146a resulted in the mitigation of systolic dysfunction. However, it did not show a complete reversal of peripartum cardiomyopathy [37].

Vascular Endothelial Growth Factor (VEGF) Agonism and Removal of Anti-Angiogenic Proteins

Apheresis has been used in order to remove circulating soluble fms-like tyrosine kinase-1 (sFlt-1) which is an antiangiogenic protein synthesized by the placenta [38]. This has been used in patients with pre-eclampsia [38]. Recently in one reported case of severe peripartum cardiomyopathy, it has been used in a patient who was on continued support with a biventricular assist device. VEGF is an angiogenic factor; its analog has been used in mouse models in order to treat peripartum cardiomyopathy [39]. However, its use in human subjects is yet to be tested.

Serelaxin

The corpus luteum, placenta, and the breast synthesize relaxin-2 which is a vasodilatory peptide that is responsible for various cardiovascular adaptations which are seen during pregnancy [40]. A recombinant formulation of relaxin-2 which is known as serelaxin has proved to be of some benefit in relieving breathlessness in cases of acute heart failure as per the findings of the RELAX-AHF trial [41]. However, it had no effect on mortality occurring due to cardiovascular causes. In a study conducted on mouse models of peripartum cardiomyopathy, relaxin led to an increase in hypertrophy of the cardiomyocyte and angiogenesis; however, it did not show any benefit in the systolic function [42]. Hence, serelaxin is an emerging treatment modality that requires further study in order to establish its complete spectrum of efficacy and safety.

Perhexiline

Perhexiline is an anti-anginal drug that has been shown to have pleiotropic effects on the metabolism of cardiac muscle [43]. Its primary mechanism of action remains the shift of metabolism to glycolysis from the fatty acids β oxidation [43]. In studies conducted in human subjects, perhexiline has shown promising results in the improvement of systolic function in cases of heart failure with reduced ejection fraction as well as providing symptomatic relief [44,45]. However, this treatment modality is yet to be studied in cases of peripartum cardiomyopathy and has certain concerns regarding drug-induced neuropathy and hepatotoxicity which need to be addressed before it can be utilized in peripartum cardiomyopathy.

Pentoxifylline

Pentoxifylline is derived from xanthine and has anti-inflammatory as well as phosphodiesterase inhibiting properties. Studies have used pentoxifylline in order to improve the systolic function of the heart and also relieve symptoms of heart failure [46]. A meta-analysis done on six trials concluded that pentoxifylline reduced all-cause mortality in heart failure patients [46]. A trial was conducted in South Africa in 30 patients suffering from peripartum cardiomyopathy; they were enrolled and treated with pentoxifylline along with conventional therapy. It was observed that the addition of pentoxifylline reduced morality in peripartum cardiomyopathy and 52% of patients in the group not receiving pentoxifylline had poor outcomes; whereas, 27% of patients in the group receiving pentoxifylline had a poor outcome [47]. Poor outcome in this study was defined as death, failure to improve the left ventricular ejection fraction of more than 10 absolute points, or failure to improve the functional class III or IV at the latest follow-up

Follow-up of patients

Multiple studies have been conducted to determine if the medications for peripartum cardiomyopathy can be tapered safely. Various studies have shown that patients with recovered left ventricle function can be safely tapered off the medication. In one study, five patients were tapered off all medications and none of them experienced any reduction in the functioning of the left ventricle on an average follow-up period of 29 months [48]. Another study reported that two patients who had complete recovery of left ventricle function had deterioration of ejection fraction following the discontinuation of their medications and this deterioration was seen at 24 and 34 months respectively following the diagnosis [49]. Two more studies concluded that women with recovering left ventricle function may experience left ventricle diastolic dysfunction as well as a reduction in exercise capacity [50], residual injury to the myocardium, and angiogenic imbalance [51]. This suggests that women who recover may benefit from long-term therapy which is guidance mediated for heart failure with reduced ejection fraction. It is essential for women with peripartum cardiomyopathy to maintain a regular follow-up with the health centers irrespective of the recovery of the left ventricle or whether their medications are discontinued.

## Conclusions

Peripartum cardiomyopathy is an important cardiovascular disease affecting the peripartum period which may lead to adverse outcomes. The peripartum period is considered the most sensitive period for the mother as well as the fetus. Therefore, special vigilance and care should be provided in order to prevent mortality due to peripartum cardiomyopathy which occurs mostly in the last month of pregnancy. Different treatment modalities are available in order to treat peripartum cardiomyopathy ranging from general considerations such as salt restriction to the use of drugs such as diuretics, β blockers, vasodilators, anticoagulants, and more recently, prolactin inhibitors. Mechanical circulatory support and cardiac transplantation may be required in severe cases refractory to medical management. A case-specific pregnancy consideration approach should be adapted in order to decide the mode and time of delivery. The mode of delivery should be decided after assessing the severity of the disease. If the disease has progressed to advanced stages or presents with any medical or obstetrical complication, then instead of vaginal delivery, lower segment cesarean section could be taken into consideration.
